# BRAIN FITNESS INTERVENTION ON KNOWLEDGE AND WORRIES ABOUT ALZHEIMER’S: A RANDOMIZED CONTROLLED TRIAL

**DOI:** 10.1093/geroni/igz038.000

**Published:** 2019-11-08

**Authors:** Kaipeng Wang, Fei Sun, Qiuling An, Yanfei Han, Yi Zhou

**Affiliations:** 1 Texas State University, San Marcos, Texas, United States; 2 Michigan State University, East Lansing, Michigan, United States; 3 East China Normal University, Putuo, Shanghai, China

## Abstract

As one of the world’s fastest aging countries, China’s growing prevalence of Alzheimer’s and related dementias (ADRD) poses concerns among older adults. Lack of knowledge about ADRD and excessive worries about ADRD can cause cumulative stress and threaten physical and psychological well-being of older adults. We conducted a randomized controlled trial to examine the effectiveness of a Brain Fitness Intervention (BFI) on the knowledge and worries about Alzheimer’s at three senior residential care facilities in China. Ninety older adults aged 60 and above underwent randomization. Fifty participants in the intervention group received eight weekly BFI sessions, including Tai Chi exercise, experiential learning, and group discussions. The primary outcomes were the changes from baseline to Week 8 in the scores on the ADRD knowledge scale (ranging 5–50) and worry scale (ranging 5–45). Twenty-seven participants withdrew from the study. Intent-to-treat analysis showed that the estimated mean change in knowledge on ADRD was 4.26 in the treatment group and -1.52 in the control group (p < 0.001). The estimated mean change in worries about ADRD was -10.25 in the treatment group and -2.9 in the control group (p < 0.001). Results remained robust in sensitivity analysis adjusting for study sites and baseline characteristics. Heterogeneity analysis showed that the treatment effect became stronger with the increase of age. Findings support the effectiveness of BFI in increasing ADRD knowledge and reducing worries among Chinese older adults. Future trials with larger sample sizes will be needed for more conclusive results.

